# Neutrophil-to-lymphocyte ratio vs C-reactive protein as early predictors of anastomotic leakage after colorectal surgery: A retrospective cohort study

**DOI:** 10.1016/j.amsu.2021.102201

**Published:** 2021-03-05

**Authors:** Diana A. Pantoja Pachajoa, Manuel Gielis, René M. Palacios Huatuco, Milagros N. Benitez, Micaela N. Avila, Alejandro M. Doniquian, Fernando A. Alvarez, Matías Parodi

**Affiliations:** General Surgery Service, Clínica Universitaria Reina Fabiola, Universidad Católica de Córdoba, Oncativo, 1248, Córdoba Capital, Argentina

**Keywords:** Neutrophil-to-lymphocyte ratio, C-reactive protein, Preclinical marker, Colorectal surgery, Complications, Anastomotic leak

## Abstract

**Introduction:**

Colorectal surgery (CRS) is associated with high morbidity rates, being anastomotic leakage (AL) one of the most serious complications with an incidence as high as 15%, accounting for up to a third of mortality in these procedures. The identification of pre-clinical markers may allow an early diagnosis and a timely intervention. The objective of the present study was to compare the performance of neutrophil-to-lymphocyte ratio (NLR) vs C-reactive protein (CRP) as early predictors of AL in CRS.

**Methods:**

A retrospectively analyzed consecutive patients who underwent a colorectal surgery with anastomosis from June 2015 to April 2019. Receiver-operating characteristic (ROC) curves were used to find the cutoff points with the best diagnostic performance of AL.

**Results:**

A total of 116 patients were included. From 43 patients (37%) who developed a total of 63 complications, 9 (7.76%) presented with an AL with a median of 8 days (range: 5–9). No significant differences were found for NLR between patients with vs without AL. In contrast, median CRP was significantly higher in patients who subsequently presented with AL, both on day 4 (164 vs 64, p = 0.04) and 5 (94 vs 44, p < 0.001) after surgery. The best predictive performance through ROC curves was found on postoperative day 5, with a CRP value of >54 mg/dL (AUC: 0.81, Sensitivity: 89%, Specificity: 61%).

**Conclusions:**

CRP appears superior to NLR as an early predictor of AL following CRS. The best diagnostic performance was obtained on postoperative day 5 with a cutoff value of >54 mg/dL.

## Highlights

• In the present study, CRP was found to be superior to NLR as an early predictor of clinically significant AL following CRS.
• The best predictive performance was obtained at postoperative day 5 with a cutoff value > 54 mg/dL.
• The importance of this finding may contribute during the hospital stay from the third postoperative day to the early diagnosis of LA.

## 1 Introduction

Colorectal surgery (CRS) has improved immensely over recent decades as a consequence of better presurgical preparation, antibiotic prophylaxis, surgical technique and postoperative management. However, it is may still be associated with somehow high morbidity and mortality rates [1]. Prospective multicenter studies have demonstrated that 30-day morbidity and mortality may go up to 35% and 9% respectively [2,3]. The most frequent postoperative complications include surgical site infection, intraabdominal abscess, ileus, hemorrhage, and anastomotic leakage (AL) [4]. Of these, AL is considered the most serious, with an incidence that varies from 3% to 15% and is responsible of up to a third of mortality in these patients. It is worth noting that most of them become evident between postoperative day 5 and 7, and the highest rates occur in coloanal anastomosis (10–20%) [5]. Early detection of serious postoperative complications such as AL in patients who undergo CRS is crucial for effective decision-making that anticipates septic complications. Although few markers have been studied as predictors of postoperative complications, in an era of enhanced recovery after surgery (ERAS) or Fast Track Surgery [6] in which patients are discharged rapidly, the identification and use of pre-clinical inflammatory biomarkers may become utmost important to allow early diagnosis of serious complications. C-reactive protein (CRP) is an acute phase reactant of hepatic synthesis, which raises in most of the inflammatory processes in response to proinflammatory cytokines as interleukin (IL) 6, IL-1 beta, tumor necrosis factor-alpha and interferon-gamma [7]. It has been noted that high concentrations of CRP at the third and fourth postoperative day may be associated to the development of septic intra-abdominal complications such as AL after CRS [8]. On the other hand, pre and postoperative neutrophil-to-lymphocyte ratio (NLR), a simple and costless marker of subclinical inflammatory response, has recently been identified as a useful predictor of major complications after surgery [9,10]. Moreover, it has been recently suggested that preoperative NLR might be better than CRP as predictor of 30-day morbidity after major abdominal surgery [11]. With regards to CRS, it has been recently suggested that an elevated preoperative NLR may be a risk factor for major surgical complications following colorectal resection, with a trend towards the occurrence of AL [12]. Although NLR may be advantageous in the clinical setting given that it has shown a faster kinetic pattern than CRP in response to surgical trauma, whether postoperative NLR can early predict the occurrence of AL in after CRS remains unknown [13].

The aim of the present study was to determine the value of postoperative NLR as early predictor of AL in patients who undergo CRS, and compare its diagnostic performance with CRP.

## 2 Material and methods

### 2.1 Study design and population

The current report represents a single-institution retrospective cohort analysis of a prospectively maintained database. Data for consecutive patients submitted to colorectal surgery from June 2015 to April 2019 at a General Surgery Service of a tertiary referral University Hospital was extracted. All colorectal resections were included in the study analysis regardless on the approach used (open or laparoscopic), the confection of a diverting loop ostomy or the timing of surgery (urgent or scheduled). From a total 152 patients, 36 were excluded due to incomplete medical records, hematological diseases, extra-abdominal infections, definitive colostomy (i.e. Miles operation), or those who did not have a primary anastomosis (e.g. Hartmann's operation).

The study was performed in accordance with the 1964 Declaration of Helsinki (revised in 2013) and whit the approval of the institutional review board of our local hospital. It was registered with ClinicalTrials.gov (NCT04673110) which can be found via the following link: https://clinicaltrials.gov/show/NCT04673110. The research has been reported in line with the STROCSS [14].

### 2.2 Surgical technique and follow-up

All the procedures were performed by staff surgeons, including a colorectal surgery specialist. Regarding the techniques used for anastomosis, all colorectal or ileorectal anastomosis were performed using staplers and ileocolic anastomosis were either performed using staplers or hand-sewn technique. After discharge, all patients were controlled through clinical evaluation and routine laboratory tests in an outpatient office at 2 weeks and 1 month after surgery, and every 4 months thereafter in oncological patients. Data on each patient was recorded prospectively from the date of index operation up to a minimum of 12 months after surgery or until death whichever occurred first.

### 2.3 Study variables and definitions

Patients demographics, indication for surgery, timing of the operation (elective or emergency), surgical risk according to ASA (American Society of Anesthesiologists), resection type, surgical approach (open or laparoscopic), need of diverting ileostomy, hospital stay and postoperative complications were analyzed. The Comprehensive Complication Index (CCI) was used to grade postoperative morbidity, which integrates in a single formula all the complications suffered by the patient according to the Clavien-Dindo score, summarizing postoperative (POP) morbidity in a grading score that goes from 1 to 100 [15]. Serum concentration of CRP and NLR during the first 5 postoperative days was recorded. All patients had complete cytology every day up to day 5 in order to calculate the ratio between the absolute value of neutrophils and the absolute value of lymphocytes. A CRP level below 5 mg/dL was considered normal. We analyzed the relationship between the variables and the development of AL. According to laboratory findings and clinical judgement, additional imaging studies were employed to rule out AL. AL was defined as suture line disruption with intestinal content leakage or abscess formation, associated with fever or abdominal pain, and confirmed by a CT-scan or re-operation up to 3 months after CRS.

### 2.4 Statistical analysis

Continuous variables are expressed as means and standard deviation (SD) for symmetrically distributed, and median (range) for non-symmetrically distributed data. Categorical variables were expressed as frequencies (percentages). The Mann-Whitney test was used for comparison of continuous variables and Chi-squared test or Fisher's exact test was used for comparisons of categorical variables between patients with and without AL. The differences between variables were considered significant at a value of p ≤ 0.05. Receiver operating characteristic (ROC) curves were used to evaluate the diagnostic accuracy of CRP and NLR as predictors of AL, determining sensitivity (Se), specificity (Sp), positive predictive value (PPV) and negative predictive value (NPV). The diagnostic accuracy of the plasmatic markers studied was based on the area under the ROC curve (AUC). We used the Youden Index to find the cutoff point with the best diagnostic performance of CRP and NLR on the first five postoperative days. SPSS version 24 and GraphPad version 7 were used for statistical analysis.

## 3 Results

**Fig. 1:**
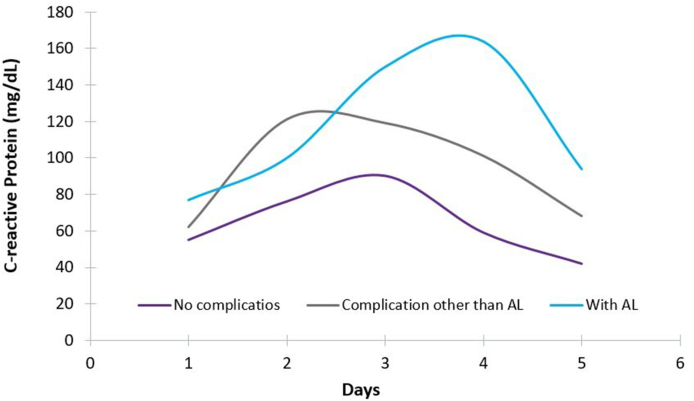
Median postoperative concentration curves of C-reactive protein (CRP) in patients with no complications, complication other than AL, and those who presented AL.

**Fig. 2:**
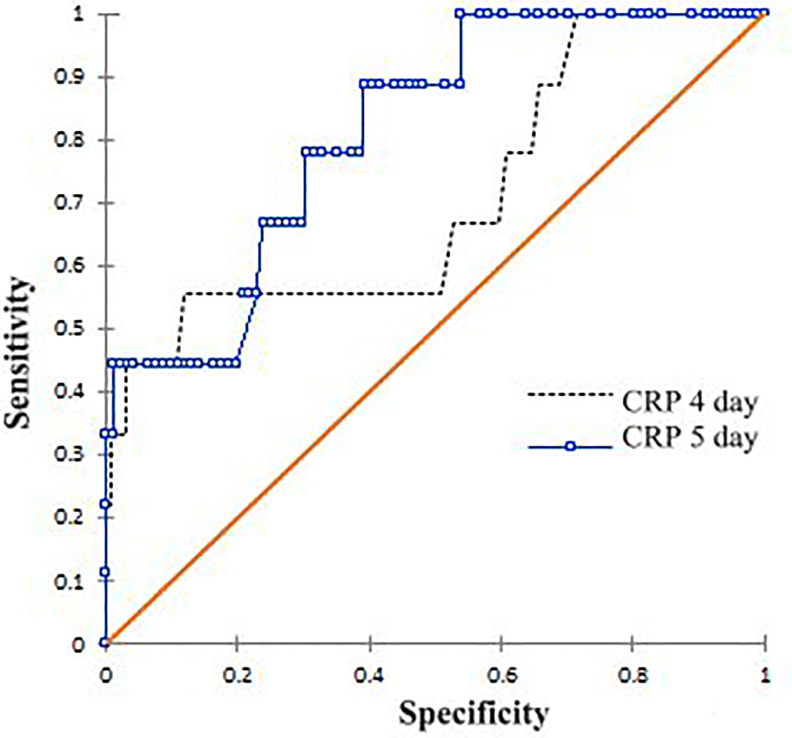
Receiver-operating characteristic (ROC) curves of C-reactive protein (CRP) at postoperative days 4 and 5 in patients with AL. AUC, area under the curve; cut-off value giving equal weight to sensitivity and specificity.

A total of 116 patients represented the study population. Fifty-two percent (n: 60) were men and the median age was 62 years (range: 19–90). The majority of the surgical procedures were elective (n: 101, 82%) and most indicated for the treatment of colorectal cancer (n: 86, 74%). Overall morbidity and mortality of the studied population was 37% and 0% (30 days/1 year) respectively. During postoperative recovery 9 patients developed AL (8%) in a median of 8 days (range: 5–9). When comparing baseline variables between patients with and without AL, only weight was found significantly higher in the AL group vs no-AL group (Table 1). Not surprisingly, patients with AL had higher morbidity according to the CCI index than those without AL (44.98 ± 14.67 vs 28.44 ± 17.11, p < 0.01) and longer median hospital stay (25 vs 8 days; p < 0.001). Regarding the studied preclinical predictors of AL, no significant differences were found for NLR between the two groups in the first 5 postoperative days (Table 1). In contrast, as shown in Fig. 1, although median CRP concentration curves tended to stabilize after postoperative day 2 and decline after postoperative day 3 in patients without complications or with complications other than AL, CRP continued to rise in those who presented AL. In fact, median CRP level was significantly higher in patients who presented AL vs no-AL at postoperative day 4 (164 vs 64, p = 0.04) and 5 (94 vs 44, p < 0.001), respectively. The best predictive performance according to ROC curves analysis was found at postoperative day 5, with a CRP value of >54 mg/dL (AUC: 0.81; 95% CI: 0.66–0.97) (Fig. 2). The likelihood of having AL at postoperative day 5 in patients with a CRP >54 mg/dL was 89%, whereas no patient with a CRP level <40 mg/dL presented AL. Table 2 summarizes the diagnostic accuracy of CRP.

**Table 1:** Demographic, clinical, and operative characteristics between the study groups.

	Patients without AL (n= 107)	Patients with AL (n=9)	p
**Gender, n (%)**			0.49
Male	54 (50.5)	6 (66.7)	
Female	53 (49.5)	3 (33.3)	
**Age, n (%), years**			0.60
>60	63 (58.9)	7 (77.8)	
<60	44 (41.1)	2 (22.2)	
**BMI, n (%), kg/m**^**2**^			0.10
< 20	4 (3.7)	0 (0)	
20,1-25	45 (42.1)	2 (22.2)	
> 25,1	58 (54.2)	7 (66.7)	
**Weight, mean ± SD, kg**	75 ± 15	88 ± 13	0.01
**ASA, n (%)**			0.17
I	27 (25.2)	0 (0)	
II	64 (59.8)	6 (66.7)	
III	15 (14)	3 (33.3)	
IV	1 (1)	0 (0)	
**Surgical opportunity, n (%)**			0.62
Emergency	13 (12.1)	2 (22.2)	
Schedule	94 (87.9)	7 (77.8)	
**Diagnostic entity, n (%)**			0.34
Oncological	77 (71.9)	9 (100)	
Inflammatory	24 (22.5)	0 (0)	
Others	6 (5.6)	0 (0)	
**Location, n (%)**			1.00
Colon	86 (80.4)	7 (77.8)	
Right	28	2	
Left	54	3	
Transverse	4	2	
Rectum	17 (15.9)	2 (22.2)	
Superior	2	1	
Medium	9	1	
Low	6	0	
Ileocecal valve	4 (3.7)	0 (0)	
**Surgical approach, n (%)**			0.49
Open	60 (56.1)	5 (55.5)	
Laparoscopic	47 (43.9)	4 (44.5)	
**Protective ostomy, n (%)**			0.47
Yes	25 (23.4)	2 (22.2)	
No	82 (76.6)	7 (77.8)	
**Hospitalization, median (range), days**	8 (4-14)	25 (8-48)	0.001
**CCI, mean ± SD**	21 ± 17,11	45± 14,67	0.01
**CRP, median (range), mg/dl**			
Day 1	58 (4 - 411)	77 (24 -260)	0.18
Day 2	88 (8 - 405)	100 (34 -248)	0.69
Day 3	91 (3-340)	150 (46 -339)	0.07
Day 4	64 (11-290)	164 (46-304)	0.04
Day 5	44 (6-230)	94 (40-330)	<0.01
**NLR, median (range)**			
Day 1	9.50 (1.90-47)	8.98 (3,5 -23)	0.70
Day 2	6.11 (1.58-42)	7.35 (3.8-15)	0.18
Day 3	4.69 (0.86-30.66)	7.64 (2.20-22.25)	0.20
Day 4	3.92 (1.38-47)	5.13 (2.16-47)	0.33
Day 5	3.88 (1.34-95)	5.00 (2.03-30)	0.15

AL, anastomotic leakage; BMI, body mass index; ASA, American society of Anesthesiologist; CCI, comprehensive complication index; CRP, C-reactive protein; NLR, neutrophil-to-lymphocyte ratio.

**Table 2:** Diagnostic Accuracy of C-reactive protein (CRP).

Day	Cutoff	AUC	95% CI	Se	Sp	PPV	NPV
**4**	63 mg/dL	0.714	0.485-0.943	67%	50%	14%	92%
**5**	54 mg/dL	0.814	0.658-0.970	89%	61%	18%	98%

AUC, area under the curve; CI, confidence interval; Se, sensibility; Sp, specificity; PPV, positive predictive value; NPV, negative predictive value.

## 4 Discussion

In the present study, focused on the role of postoperative NLR in predicting AL after CRS, it was found that NLR was unable to predict AL during the first 5 postoperative days. On the contrary, a CRP of >54 mg/dL at postoperative day 5 was found to best predict clinically significant AL in patients submitted to CRS.

Postoperative complications have negative effects on cumulative morbidity and mortality after surgery. In this study, an AL incidence in CRS of 7.76% (n = 9) was found to be similar to previous reports (7.6–9.6%) [16,17]. Not surprisingly, those patients who developed AL had 2 times higher cumulative morbidity by CCI score (45 vs 21; p < 0.01) and a 3 times longer median hospital stay (25 vs 8 days; p < 0.001) than those without AL. The preoperative utilization of risk scores based on known risk factors of postoperative complications in high-complexity procedures such as CLS is of great utility to early stratify patient risk which has a positive impact on outcomes. Several factors have been independently associated with increased risk of AL after CRS, such as advanced age, male sex, obesity, ASA risk >2, smoking, diabetes, preoperative serum albumin <4 g/dL, thrombocytosis, emergency surgery, rectal cancer, preoperative radiation, chronic immunosuppressive medications [18,19]. In our study, only median weight was found to be significantly higher in patients with AL compared with those without AL (88 vs 75 kg; p < 0.01), but this could well be explained by a type 2 statistical error for many of the other studied variables. Although this data undoubtedly helps to preoperatively identify high-risk patients for primary prevention of AL and deciding whether or not to create a diversion stoma to reduce its clinical impact, none can early diagnose AL once the surgery has been undertaken. Therefore, despite proper patient selection and correct surgical technique to avoid AL, the use of postoperative predictive serum biomarkers could become of utmost importance in daily practice to help preclinical diagnosis of AL and guide decision-making for secondary prevention and early therapeutical management of AL after CRS.

Forget et al. [11] found a higher NLR at postoperative day 7 in patients with postoperative complications (10.73 vs 4.73; p < 0.001), being the only independent variable associated with major complications in the multivariate analysis. Although this finding is of interest, we consider that for a biomarker to be useful from the clinical viewpoint, it should be able to predict complications earlier in the recovery period, since in the nowadays context of ERAS or Fast-Track protocols, the patient is already out of the hospital by postoperative day 7 and most AL are already clinically manifest. In the present study we could not find significant differences for NLR between patients with and without AL up to postoperative day 5. It has been noted that high concentrations of CRP at third and fourth postoperative day may be associated to the development of septic intra-abdominal complications after CRS, and its predictive ability does not seem to be impacted by the surgical approach, although it has been demonstrated that patients who undergo a minimally invasive approach have a lower immune response and incidence of complications than those who undergo an open surgery [20]. Ramanathan et al. [21] concluded in a study including 344 patients that although the magnitude of the systemic inflammatory response is greater following open surgery compared with laparoscopic resection, CRP threshold predictive of postoperative infective complications were similar on postoperative days 3 (180 mg/dL) and 4 (140 mg/dL) following both open and laparoscopic resection for colon cancer. In line with this results, the present study demonstrates that the magnitude of the systemic inflammatory response, particularly the serum concentration of CRP determined at postoperative day 4 and 5, is significantly higher in patients who developed an AL, with median values of 164 mg/dL (p = 0.04) and 94 mg/dL (p < 0.01), respectively. Although there was also a clear trend of higher median CRP at postoperative day 3 in patients with AL (150 vs 91 mg/dL; p = 0.07), this difference did not reach statistical significance most likely due to a type 2 statistical error. Our results are consistent with previous studies, as that of Matthiessen et al. [22] who observed that patients who suffered AL after an anterior rectal resection had persistently higher CRP levels from postoperative day 2 compared to patients who did not develop AL, but without defining a predictive cutoff value. Later, Platt et al. [23] recommend that a CRP value > 170 mg/dL at postoperative day 3 was useful for detecting infectious complications after a curative resection of colorectal cancer. On the other hand, Ortega-Deballon et al. [24] suggested avoiding discharge at postoperative day 4 if a CRP value > 125 mg/dL occurs. Although the results of the previous studies differ slightly, all of them agree that CRP value has a significant clinical potential in the prediction or exclusion of infectious complications in a sufficiently early stage to facilitate timely interventions. When evaluating the diagnostic accuracy of CRP the present study, although we obtained an AUC of 0.714 for postoperative day 4, the best performance was found at postoperative day 5 with an AUC of 0.814 and a cutoff value in 54 mg/dL (Se: 89%, Sp: 61%, PPV: 18%, NPV: 98%). Therefore, taking these results into account, we suggest routine CRP monitoring from postoperative day 3 after CRS, and if an infectious complication is suspected due to a CRP level >54 mg/dL that does not decline in the following 48 h s, this should prompt a thorough clinical examination, along with laboratory tests and imaging studies in order to rule-out AL, even in asymptomatic patients before discharge.

Despite its clear limitations, such us being of retrospective nature, the somehow low number of patients included, and the heterogeneity of the studied population, this study is the first to evaluate whether postoperative NLR con predict AL after CRS, and compare its efficacy against that of an already known serum marker such as CRP.

## 5 Conclusion

In the present study CRP was found to be superior to NLR as an early predictor of clinically significant AL following CRS. The best predictive performance was obtained at postoperative day 5 with a cutoff value > 54 mg/dL. Even though further studies are needed to confirm a higher predictive accuracy for the CRP compared NLR, we encourage the routine use of this complementary, readily available, noninvasive low-cost examination in institutions performing CRS as an effort to continue improving patient safety. Its routine use during hospital stay from postoperative day 3 may contribute to early diagnose AL and effective decision-making, such us delaying patient discharge, performing a computed tomography or even supporting laparoscopic surgical exploration in oligosymptomatic patients, all of which are crucial to anticipate septic compromise and prevent cumulative morbidity in this patients.

## Funding

No source to be stated.

## Informed consent

Informed consent was obtained from all individual participants included in the study.

## Declaration of competing interest

The authors declare that they have no conflict of interest.

## References

[1] Paun B.C., Cassie S., MacLean A.R., Dixon E., Buie W.D. (2010). Postoperative complications following surgery for rectal cancer. Ann. Surg..

[2] Alves A., Panis Y., Mathieu P., Mantion G., Kwiatkowski F., Slim K. (2005). Postoperative mortality and morbidity in French patients undergoing colorectal surgery: results of a prospective multicenter study. Arch. Surg..

[3] Leung E., Ferjani A.M., Stellard N., Wong L.S. (2009). Predicting post-operative mortality in patients undergoing colorectal surgery using P-POSSUM and CR-POSSUM scores: a prospective study. Int. J. Colorectal Dis..

[4] Choy P.Y.G., Bissett I.P., Docherty J.G., Parry B.R., Merrie A., Fitzgerald A. (2011). Stapled versus handsewn methods for ileocolic anastomoses. Cochrane Database Syst. Rev..

[5] Kingham T.P., Pachter H.L. (2009). Colonic anastomotic leak: risk factors, diagnosis, and treatment. J. Am. Coll. Surg..

[6] Kehlet H. (2005). Fast-track colonic surgery: status and perspectives. Recent Res. Can. Res..

[7] Gauldie J., Richards C., Harnish D., Lansdorp P., Baumann H. (1987). Interferon beta 2/B-cell stimulatory factor type 2 shares identity with monocyte-derived hepatocyte-stimulating factor and regulates the major acute phase protein response in liver cells. Proc. Natl. Acad. Sci. U. S. A.

[8] Singh P.P., Zeng I.S.L., Srinivasa S., Lemanu D.P., Connolly A.B., Hill A.G. (2014). Systematic review and meta-analysis of use of serum C-reactive protein levels to predict anastomotic leak after colorectal surgery. Br. J. Surg..

[9] Da Silva M., Cleghorn M.C., Elnahas A., Jackson T.D., Okrainec A., Quereshy F.A. (2017). Postoperative day one neutrophil-to-lymphocyte ratio as a predictor of 30-day outcomes in bariatric surgery patients. Surg. Endosc..

[10] Mohri Y., Tanaka K., Toiyama Y., Ohi M., Yasuda H., Inoue Y. (2016). Impact of preoperative neutrophil to lymphocyte ratio and postoperative infectious complications on survival after curative gastrectomy for gastric cancer: a single institutional cohort study. Medicine (Baltimore).

[11] Forget P., Dinant V., De Kock M. (2015). Is the neutrophil-to-lymphocyte ratio more correlated than C-reactive protein with postoperative complications after major abdominal surgery?. PeerJ.

[12] Josse J.M., Cleghorn M.C., Ramji K.M., Jiang H., Elnahas A., Jackson T.D. (2016). The neutrophil-to-lymphocyte ratio predicts major perioperative complications in patients undergoing colorectal surgery. Colorectal Dis..

[13] Wasko M.K., Struminski M., Bobecka-Wesolowska K., Kowalczewski J. (2017). Neutrophil-to-lymphocyte ratio shows faster changing kinetics than C-reactive protein after total hip and knee arthroplasty. J. Orthop. Transl..

[14] Agha R., Abdall-Razak A., Crossley E., Dowlut N., Iosifidis C., Mathew G., for the STROCSS Group (2019). The STROCSS 2019 guideline: strengthening the reporting of cohort studies in surgery. Int. J. Surg..

[15] Slankamenac K., Graf R., Barkun J., Puhan M.A., Clavien P.A. (2013). The comprehensive complication index: a novel continuous scale to measure surgical morbidity. Ann. Surg..

[16] Yu Y., Wu Z., Shen Z., Cao Y. (2020). Preoperative C-Reactive Protein-To-Albumin Ratio Predicts Anastomotic Leak in Elderly Patients after Curative Colorectal Surgery.

[17] Trencheva K., Morrissey K.P., Wells M., Mancuso C.A., Lee S.W., Sonoda T. (2013). Identifying important predictors for anastomotic leak after colon and rectal resection: prospective study on 616 patients. Ann. Surg..

[18] Park J.S., Choi G.S., Kim S.H., Kim H.R., Kim N.K., Lee K.Y. (2013). Multicenter analysis of risk factors for anastomotic leakage after laparoscopic rectal cancer excision: the Korean laparoscopic colorectal surgery study group. Ann. Surg..

[19] Choi H.K., Law W.L., Ho J.W.C. (2006). Leakage after resection and intraperitoneal anastomosis for colorectal malignancy: analysis of risk factors. Dis. Colon Rectum.

[20] Shibata J., Ishihara S., Tada N., Kawai K., Tsuno N.H., Yamaguchi H. (2015). Surgical stress response after colorectal resection: a comparison of robotic, laparoscopic, and open surgery. Tech. Coloproctol..

[21] Ramanathan M.L., MacKay G., Platt J., Horgan P.G., McMillan D.C. (2015). The impact of open versus laparoscopic resection for colon cancer on C-reactive protein concentrations as a predictor of postoperative infective complications. Ann. Surg Oncol..

[22] Matthiessen P., Henriksson M., Hallböök O., Grunditz E., Norén B., Arbman G. (2008). Increase of serum C-reactive protein is an early indicator of subsequent symptomatic anastomotic leakage after anterior resection, Color. Dis.

[23] Platt J.J., Ramanathan M.L., Crosbie R.A., Anderson J.H., McKee R.F., Horgan P.G. (2012). C-reactive protein as a predictor of postoperative infective complications after curative resection in patients with colorectal cancer. Ann. Surg Oncol..

[24] Ortega-Deballon P., Radais F., Facy O., D’Athis P., Masson D., Charles P.E. (2010). C-reactive protein is an early predictor of septic complications after elective colorectal surgery. World J. Surg..

